# Pro-Senescence and Anti-Senescence Mechanisms of Cardiovascular Aging: Cardiac MicroRNA Regulation of Longevity Drug-Induced Autophagy

**DOI:** 10.3389/fphar.2020.00774

**Published:** 2020-05-26

**Authors:** Lakshmi Pulakat, Howard H. Chen

**Affiliations:** ^1^Molecular Cardiology Research Institute, Tufts Medical Center, Boston, MA, United States; ^2^Department of Medicine, Tufts University School of Medicine, Boston, MA, United States

**Keywords:** cardiovascular, aging, senescence, autophagy, microRNA, longevity drugs, caloric restriction

## Abstract

Chronological aging as well as biological aging accelerated by various pathologies such as diabetes and obesity contribute to cardiovascular aging, and structural and functional tissue damage of the heart and vasculature. Cardiovascular aging in humans is characterized by structural pathologic remodeling including cardiac and vascular fibrosis, hypertrophy, stiffness, micro- and macro-circulatory impairment, left ventricular diastolic dysfunction precipitating heart failure with either reduced or preserved ejection fraction, and cardiovascular cell death. Cellular senescence, an important hallmark of aging, is a critical factor that impairs repair and regeneration of damaged cells in cardiovascular tissues whereas autophagy, an intracellular catabolic process is an essential inherent mechanism that removes senescent cells throughout life time in all tissues. Several recent reviews have highlighted the fact that all longevity treatment paradigms to mitigate progression of aging-related pathologies converge in induction of autophagy, activation of AMP kinase (AMPK) and Sirtuin pathway, and inhibition of mechanistic target of rapamycin (mTOR). These longevity treatments include health style changes such as caloric restriction, and drug treatments using rapamycin, the first FDA-approved longevity drug, as well as other experimental longevity drugs such as metformin, rapamycin, aspirin, and resveratrol. However, in the heart tissue, autophagy induction has to be tightly regulated since evidence show excessive autophagy results in cardiomyopathy and heart failure. Here we discuss emerging evidence for microRNA-mediated tight regulation of autophagy in the heart in response to treatment with rapamycin, and novel approaches to monitor autophagy progression in a temporal manner to diagnose and regulate autophagy induction by longevity treatments.

## Introduction

Global deaths from cardiovascular disease increased by 41% between 1990 and 2013 and population ageing alone contributed to an estimated 52.5% increase in these deaths (Roth et al., 2015; Roth et al., 2018). The 2019 United Nations statement predicts that by 2050, one in six people in the world will be over the age of 65, up from one in 11 in 2019 (United Nations, 2019). Older adults are particularly susceptible to cardiovascular diseases (CVD), a major contributor to frailty and mortality. The incidence of CVD is ~75% in those at the age of 60–79 years, and increases to ~86% in those above the age of 80 (Appelman et al., 2015; Childs et al., 2018; Roth et al., 2018; Rodgers et al., 2019; Noale et al., 2020; Stojanovic et al., 2020). Therefore, aging is both a driver and an independent risk factor for CVD.

Conversely, CVDs resulting from a variety of metabolic and environmental stressors or infections promote cellular senescence and accelerate cellular, organ-level, and organismal aging. Two well-established and preventable factors that accelerate biological aging independent of chronological age are obesity and type 2 diabetes. Currently 1.9 billion adults are overweight and 650 million people are obese (World Health Organization, 2018). Age-adjusted prevalence of obesity is 42.4% in the US and obesity affects 13.7 million children and adolescents (Hales et al., 2020). Obesity promotes diabetes—globally, an estimated 463 million adults have diabetes, a prevalence rate of one in 11 (International Diabetes Association, 2019). Moreover, newly diagnosed type 1 and type 2 diabetes cases have increased among the youth in US. Diabetes is an independent risk factor for CVD (National Diabetes Statistics Report, 2020). A report using the Swedish National Diabetes Registry comprised 318,083 patients with T2DM (1998–2012) and age-, sex-, and country-matched healthy <1.6 million controls shows that patients diagnosed of type 2 diabetes mellitus (T2DM) at ≤40 years had the highest excess risk for CVD and death. Thus, the younger obese and/or diabetic patients have increased cardiovascular risk and accelerated cardiovascular and biological aging despite their chronological young age.

## Pro-Senescence Mechanisms of Cardiovascular Aging

Cardiovascular aging in humans is characterized by structural pathologic remodeling including fibrosis, hypertrophy, loss of capillary density, macro- and micro-circulatory impairment, and cardiomyocyte death. Left ventricular diastolic dysfunction that precipitates heart failure with reduced or preserved ejection fraction, vascular fibrosis, stiffness, and impairment of synthesis and/or secretion of endothelium-derived vasoactive molecules that underlie atherosclerosis and other vasculopathies mark cardiovascular aging. These cardiovascular pathologies are common to both chronological aging and accelerated biological aging induced by metabolic diseases such as diabetes and obesity despite the differences in the stressors in these different conditions (Ghebre et al., 2016; Upadhya and Kitzman, 2017; Ungvari et al., 2018; Jin, 2019; Wagner and Dimmeler, 2020). Cellular senescence, an important hallmark of aging, is a critical factor that impairs repair and regeneration of damaged cells in cardiovascular tissues (Childs et al., 2018; Lewis-McDougall et al., 2019).Cellular senescence is a stress-responsive process that is activated by multiple stressors including elevation of reactive oxygen species, pro-inflammatory cytokines, and metabolic, mechanical, and chemical toxicity such as glucotoxicity, lipotoxicity, and chemotoxicity (Colavitti and Finkel, 2005; Matsui-Hirai et al., 2011; Childs et al., 2018; Olivieri et al., 2018; Trayssac et al., 2018; Lewis-McDougall et al., 2019). Senescent cells release senescence-associated secretory phenotype (SASP) comprised of pro-inflammatory cytokines, chemokines, extracellular matrix degrading proteins, and other deleterious factors that disrupt tissue structure and function. Importantly, a relatively low abundance of senescent cells (10%) is sufficient to spread cellular senescence to surrounding cells in a tissue as well as to other tissues *via* SASP, and in conditions of diabetes, activation of DNA damage response (DDR) in cells induce senescence and thus activate a ‘malignant positive feedback loop' in which diabetes-DDR-cellular senescence further increases tissue damage and spreading of senescence (Tchkonia et al., 2013; Palmer et al., 2015; McHugh and Gil, 2018). Cellular senescence is also intrinsically linked to other hallmarks of aging such as telomere attrition, genomic instability, mitochondrial dysfunction, de-regulated nutrient sensing, stem cell exhaustion, loss of proteostasis, epigenetic alterations, and impaired intracellular communication (Lopez-Otin et al., 2013; Lidzbarsky et al., 2018). Therefore, extensive research is currently focused on understanding natural cellular processes that attenuate cellular senescence and remove senescent cells to prevent spreading of cellular senescence, and increasing these processes to mitigate aging-associated cardiovascular diseases *via* life style changes and/or longevity drugs.

## Anti-Senescence Treatments for Cardiovascular Aging

Pre-clinical and clinical evidence show that caloric restriction (CR) is an effective method to ameliorate cardiovascular pathologic remodeling and to improve cardiovascular function (Ravussin et al., 2015; de Lucia et al., 2018; Han et al., 2018; Hansen et al., 2018; Sandesara and Sperling, 2018). For example, in a rat model for myocardial infarction and post-ischemic heart failure, 1-year long CR mitigated pathologic left ventricular remodeling and improved cardiac function and inotropic reserve. CR also improved sympathetic cardiac innervation and β-adrenergic receptor levels (de Lucia et al., 2018). An average 11% CR for a 2-year period reduced cardiometabolic risk factors and increased predictors of health span and longevity in a healthy human clinical trial (Ravussin et al., 2015). CR-induced increase in β-hydroxybutyrate (β-HB) is implicated in preventing vascular senescence (Han et al., 2018). However, CR is a life style change and it is difficult to achieve patient compliance.

One of the underlying mechanisms for the anti-aging effect of CR is induction of autophagy, a process that removes senescent cells from tissues and thus prevent spreading of cellular senescence. It is now well established that autophagy is a converging point for the beneficial effects of longevity drugs such as rapamycin, other rapalogs, metformin, and resveratrol (Schiattarella and Hill, 2016; Hansen et al., 2018; Tian et al., 2019). Optimal levels of autophagy is an evolutionarily-conserved intracellular catabolism process essential to preserve cellular homeostasis in response to the same or similar stressors that induce cellular senescence. Therefore, therapeutic targeting of autophagy can be an effective approach to mitigate cardiovascular diseases (Schiattarella and Hill, 2016). In particular, cardiomyopathy caused by diabetes involves extensive deregulation of cardiac mitochondrial function and induction of mitochondrial autophagy (mitophagy) that may start as a survival mechanism, but can cause cell death when excessive (Lorenzo et al., 2013). Moreover, high glucose causes excessive mitochondrial fission that results in reactive oxygen bursts, increase in mitochondrial permeability transition pore (mPTP) opening that leads to apoptosis and Sirtuin 1 (Sirt1) inhibits process (Qin et al., 2019). Rapamycin is the first and only FDA-approved longevity drug. Metformin is an emerging longevity drug that was originally developed as an anti-glycemic drug. Resveratrol and aspirin are also drugs that are shown to have anti-aging properties. A common mode of action for all these drugs as well as CR involves inhibition of mechanistic target of rapamycin (mTOR) complex 1 (mTORC1) and activation of AMP-dependent kinase (AMPK) and Sirt1 that result in the activation of autophagy process (Rubinsztein et al., 2011; Gelino et al., 2016; Golbidi et al., 2017). The mTOR can form either mTORC1 by partnering with Raptor that activates p70S6Kinase (s6K1) or mTOR Complex 2 (mTORC2) by partnering with Rictor in a Sin1 dependent manner that activates Akt (Saxton and Sabatini, 2017). Importantly, loss of mTORC2 enhances autophagy/mitophagy (Aspernig et al., 2019).

## Autophagy Regulation and Dysregulation in CVD

Autophagy encompasses highly regulated cellular processes to maintain cellular homeostasis and proteostasis, and eliminates potentially harmful cellular stressors that induce cell death (Levine, 2005; Fernandez et al., 2018; Li et al., 2018). The highly-conserved autophagy machinery forms double-membraned autophagosomes to sequester portions of the cytoplasm and organelles, and trafficks these autophagosomes to lysosomes for degradation (Levine and Klionsky, 2004; Gatica et al., 2015; Miyamoto, 2019). Various forms of autophagy including macroautophagy, microautophagy, and chaperone-mediated autophagy all lead to turnover of intracellular components. Autophagy starts with the formation of phagophore comprised of several autophagy-related gene products (Atg1–Atg12) and other proteins that are organized in at least five molecular components: (1) the Atg1/unc-51-like kinase (ULK) complex; (2) the Beclin 1/class III phosphatidylinositol 3-kinase (PI3K) complex; (3) Atg9 and vacuole membrane protein 1 (VMP1); (4) two ubiquitin-like protein (Atg12 and Atg8/LC3) conjugation systems; and (5) proteins that mediate fusion between autophagosomes and lysosomes (Kroemer et al., 2010). Detailed mechanisms of autophagy process have been described in other reviews and not repeated here (Kroemer et al., 2010; Wang et al., 2019). While autophagy is a catabolic process that degrades damaged organelles, misfolded proteins and other harmful stressors, it also generates new building blocks (for example amino acids), energy for anabolism in conditions of nutrient deprivation, and promotes self-renewal and differentiation of pluripotent stem cells which is essential for repair of damaged tissue (Chen et al., 2018). Autophagy dysregulation tilts the balance from autophagy being the protective mechanism to exerting detrimental effects on cells leading to apoptosis, to whole-organ dysfunction, and organismal demise. Therefore, better understanding of the underlying molecular mechanisms of therapeutic induction of autophagy is of utmost importance, and the levels of autophagy need to be carefully monitored.

While basal level of autophagy is required to maintain normal cardiac function, autophagy becomes downregulated in the heart during the course of aging (Taneike et al., 2010). Age-induced impairment of autophagy further contributes to cardiac diseases (Madeo et al., 2015). For instance, mutations in ATG4C gene is associated with increased risk of heart disease in elderly patients and eventual death (Walter et al., 2011). Cardiomyocytes show age-related changes in the proteostasis pathways, resulting in calcium homeostasis impairment, reactive oxygen species induction, hypertrophy and fibrosis, and eventual structure damage and diminished cardiac function (Klionsky et al., 2016; Li et al., 2018). Cardiomyocyte-specific deletion of GSK-3α in mice accelerated cardiac aging and reduced basal autophagy levels (Zhou and Force, 2013; Zhou et al., 2013). Impaired autophagy with age slows the turnover of damaged proteasomes and contribute to age-associated CVDs and cardiomyocyte senescence (Korolchuk et al., 2009). Degradation of mitochondria by autophagy (mitophagy) is impaired in aged mice, and the induction of mitophagy improved mitochondrial function and reduced arterial wall stiffness (LaRocca et al., 2014; Palikaras et al., 2015).

Life-extending CR intervention modulates autophagy through multiple upstream regulators of autophagy, including Sirt1, AMPK, and mTOR (Rubinsztein et al., 2011; Gelino et al., 2016; Golbidi et al., 2017; Han et al., 2018; Hansen et al., 2018). The AMPK activator metformin has been shown to attenuate cardiomyocyte contractile defects in an aging-induced myocardial contractile dysfunction model (Burkewitz et al., 2016; Garg et al., 2017). Direct suppression of mTOR Complex 1 (mTORC1) *via* administration of rapamycin inhibits the adverse effects of aging, increases lifespan, and promotes autophagy in the heart as well as in many other cell types and organs, even when autophagy was suppressed by aging (Harrison et al., 2009; Bjedov et al., 2010). Supplementation with spermidine, a natural polyamine, has shown cardiac protective effects and lifespan extension by enhancing autophagy in aging-related skeletal muscle atrophy in young and old mice (Eisenberg et al., 2016; Fan et al., 2017).

Above studies are testimonials for efficacy of longevity drugs and indicate that suppression of autophagy contributes to cellular senescence and enhancing autophagy renders anti-senescence effects and mitigate cardiovascular aging. However, emerging evidence indicate that CVD-promoting pathologies such as glucotoxicity and lipotoxicity that accelerate cardiovascular aging actually increase autophagy in cardiac tissue and cause cardiac damage. Diabetes causes progressive cardiomyopathy and excessive autophagy in human heart as evidenced by increase in the levels of autophagy marker LC3B-II and its mediator Beclin-1 and decreased expression of p62, which incorporates into autophagosomes to be efficiently degraded (Munasinghe et al., 2016). Moreover, palmitic acid-induced insulin resistance is accompanied by excessive autophagy that causes cardiomyocyte death in rat H9c2 cardiomyoblast model system (Li et al., 2017). Moreover, during severe ischemia, induction of excessive autophagy worsens cardiomyocyte death and heart failure (Li et al., 2018; Liu et al., 2018; Xiao et al., 2018). Studies on cancer cells have shown that autophagy can have a pro-senescence effect because autophagy-induced increase in availability of amino acids can contribute to protein synthesis for SASP that spreads senescence (Young et al., 2009). The TOR-autophagy spatial coupling compartment (TASCC) responsible for the protein synthesis of some SASP factors serves as the platform for coupling autophagy and senescence since at TASCC autophagy supplies amino acids to activate mTOR which is involved in the synthesis of interleukins 6 and 8 that are part of SASP (Narita et al., 2011).

**Figure 1:**
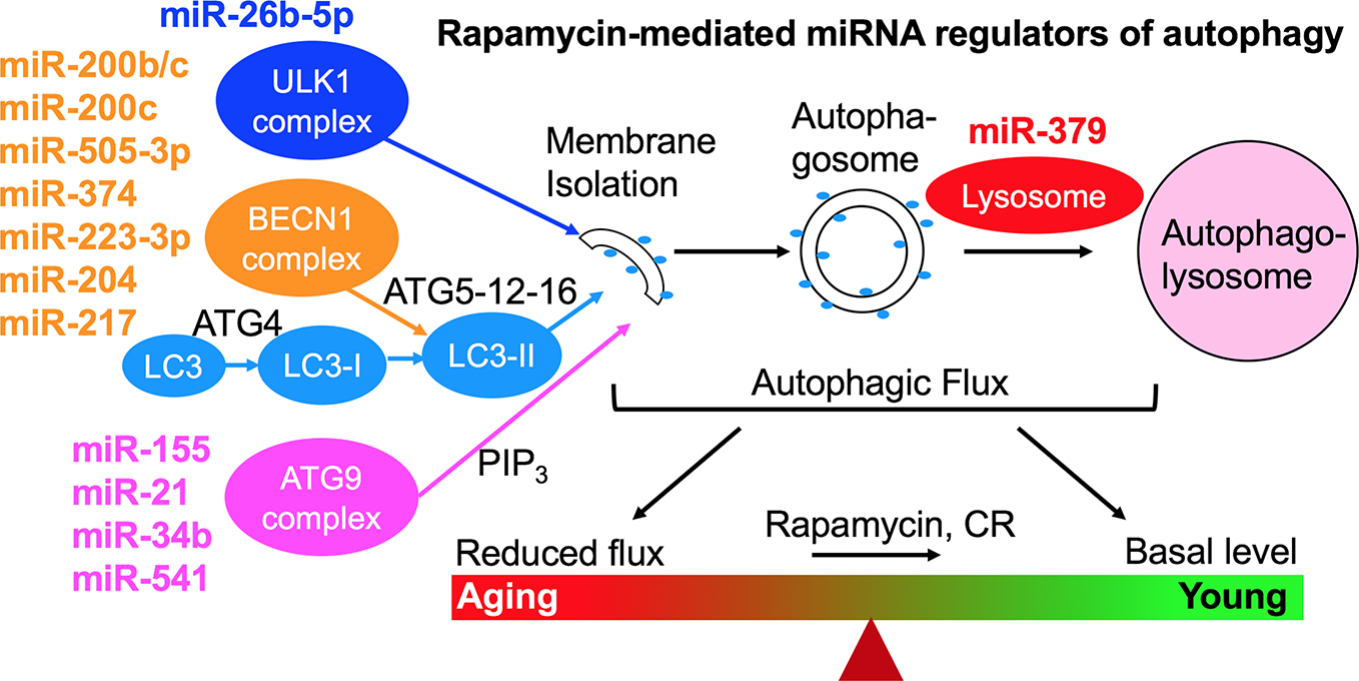
Rapamycin-induced cardiac miRNA regulation of autophagy machinery. Since these same miRNAs are also differentially expressed in response to diabetes, this autophagy-inhibiting miRNA network is an inherent cardiac mechanism in response to conditions that induce activation of autophagy. This autophagy-inhibiting miRNA network may be an adaptive mechanism to prevent excessive autophagy that leads to cardiomyopathy and heart failure, or a detrimental mechanism that prevents autophagy progression and promotes cardiac senescence. It is important to note that careful monitoring of this autophagy regulation by determining changes in cardiac autophagic flux is a critical step in evaluating whether a longevity drug- or life style-induced increase in induction of autophagy is progressing appropriately to protect the heart tissue. New non-invasive imaging approaches need to be developed to couple cardiac autophagy progression with cardiac function.

**Figure 2:**
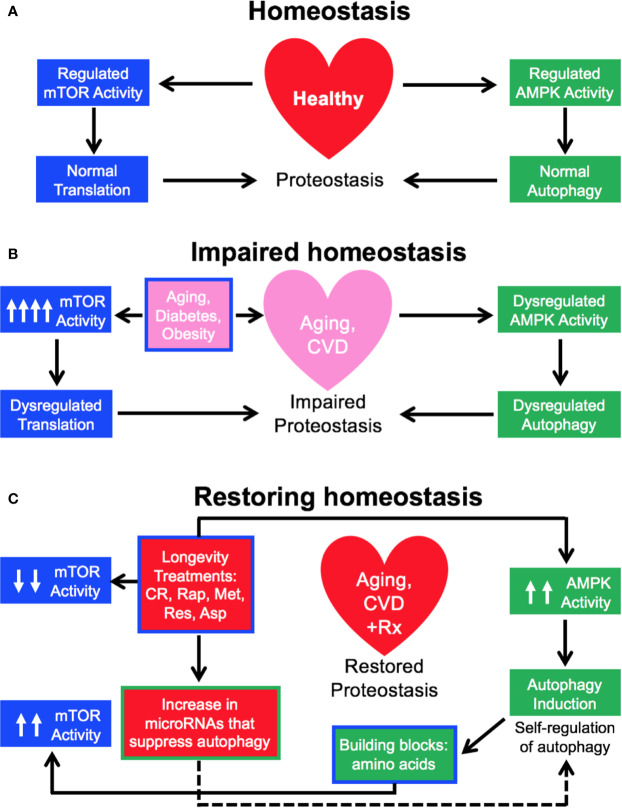
Tight Regulation of cardiac autophagy—inherent mechanisms and pathways. **(A, B)** In healthy individuals, tightly regulated activity of mTOR and AMP kinase (AMPK) maintains normal proteostasis, however, factors that accelerate cardiovascular aging also dysregulates mTOR and AMPK activity and autophagy. **(C)** Longevity treatments induce autophagy in the heart *via* initial inhibition of mTOR and activation of AMPK. However, building blocks such as amino acids generated by autophagy can reactivate mTOR, whereas an increase in microRNAs that inhibit various component proteins of autophagy machinery (shown in **Figure 1**) can inhibit further progression of autophagy in the heart.

We have studied the effect of a 3-month treatment with a comparatively low dose of rapamycin (750 μg/kg/d delivered subcutaneously) on the heart tissues of male healthy Zucker lean (ZL) and obese, diabetic Zucker obese (ZO) rats that exhibit accelerated aging, and reported that while rapamycin treatment was beneficial in reducing body weight and suppressing fasting triglycerides, insulin, and uric acid in ZO rats, it also increased blood glucose levels. In ZO rats, rapamycin initially improved multiple diastolic parameters (E/E′, E′/A′, E/Vp), but by the end of treatment these beneficial effects were reversed. In ZL rats, rapamycin increased fibrosis and induced differential regulation of intracardiac cytokines (Luck et al., 2017). A cardiac microRNA transcriptome analysis (Narita et al., 2011) showed that 40 differentially expressed miRNAs by minimum 1.5-fold in saline treated ZO rats (ZO-C) versus saline treated ZL rats (ZL-C; p ≤ 0.05) were identical to the miRNAs that were differentially expressed between ZL-C and rapamycin-treated ZL (ZL-Rap) rats (Belenchia et al., 2018). Interestingly, most of these miRNAs that exhibited increased expression in both rapamycin-treated ZL rats and saline treated diabetic ZO rats are established suppressors of autophagy (**Table 1**). Since these miRNAs are increased in rapamycin-treated healthy ZL rats, induction of such a cardiac miRNA network indicates a pan-post-transcriptional regulation of autophagy promoting genes after induction of autophagy by an established autophagy inducer and longevity drug such as rapamycin. This can be a cardiac adaptive mechanism to modulate excessive autophagy that causes cardiomyopathy in response to continuous mTORC1 inhibition by 3-month rapamycin treatment. Alternatively, since these miRNAs (**Table 1**) show increased expression in saline-treated, obese and diabetic ZO rats compared to their age-matched healthy ZL rats, activation of this autophagy-inhibiting cardiac miRNA network could be a cardiac mechanism underlying the increasing cellular senescence induced by diabetes. **Figure 1** shows the roles of autophagy regulating proteins in the autophagy machinery that are directly affected by cardiac miRNAs that are modulated similarly by Rapamycin treatment and diabetes. The fact that autophagy-inhibiting cardiac miRNA network (**Figure 1**) is activated in two independent cardiac autophagy-inducing conditions, namely diabetes and rapamycin treatment, suggest that induction of such a cardiac miRNA network is an inherent part of regulation of cardiac autophagy. Regulation of cardiac autophagy by miRNAs has been described in other CVDS as well (Sun et al., 2018), **Figure 2** shows the complex interplay of mTOR and AMPK in health and disease that modulate autophagy, and the down-stream effects of autophagy or autophagy-inducing drugs (rapamycin) that further modulate autophagy. Specifically, autophagy generates amino acids that re-activate mTOR signaling whereas autophagy-regulating miRNAs suppress progression of autophagy (**Figure 2**). Induction of autophagy by either longevity promoting pharmacological treatment (rapamycin) or pathology (diabetes) in the heart is thus intrinsically opposed by other post-transcriptional mechanisms such as increase in thirteen autophagy-inhibiting microRNAs (**Table 1**). Only three of the miRNAs in this group were enhancers of autophagy. This observation highlights the fact that although rapamycin, a drug that is considered as CR mimetic, is an activator of autophagy (consistent with the increase in autophagy enhancing miRNAs as shown in **Table 1**), the exact progression of autophagy is highly regulated by post-transcriptional mechanisms such as autophagy-inhibiting cardiac miRNA networks (**Table 1** and **Figure 2**). Since autophagy induction can be either protective or detrimental to cardiovascular tissues, effective methods to measure autophagic flux during disease progression in a temporal manner is critical.

**Table 1:** Rapamycin-induced cardiac miRNAs in 19-wk old healthy rats that modulate autophagy.

MicroRNA	Fold change in Rap-treated healthy rat heart	Effect on autophagy	Model system	Mechanism	Reference
miR-155	4.56	Enhancer	Human umbilical vein endothelial cells; cervical cancer; human and mouse septic lung injury	Inhibition of Pdk1 or growth factor-β-activated kinase-1-binding protein 2 (Tab2)	(Liu et al., 2017; Zhang et al., 2017; Wang F. et al., 2018)
miR-200b/c	4.99/2.83	Suppressor	Suppressor in Human lung adenocarcinoma and breast cancer cells.	Suppression *via* inhibition of Atg12 conjugation. Other targets are ZEB1 and UBQLN1	(Pan et al., 2015; Sun et al., 2015)
miR-200c	2.83	Enhancer	PC-3 cells	Increases microtubule-associated protein 1A/1B-LC3-II *via* endoplasmic reticulum stress mediator IRE1α	(Sohn, 2018)
miR-21	5.28	Suppressor	NRK-52E cells, rat ischemia/reperfusion model, nucleus pulposus cells, non-small cell lung cancer cells	Inhibits Ras-related protein Rab11A and PTEN	(Liu et al., 2013; Liu et al., 2015; Wang W. et al., 2018)
miR-26b-5p	5.13	Suppressor	Rat model for exercise induced left ventricular hypertrophy and rat H9c2 cardiomyoblasts	Inhibits ULK1	(Qi et al., 2020)
miR-411	7.16	Suppressor	Chondrocytes	Inhibits HIF-1α	(Yang et al., 2020)
miR-301a	4.86	Suppressor	Prostate cancer cells in hypoxia	Inhibits NDRG2	(Wang et al., 2016)
miR-505-3p	4.92	Suppressor	Mouse cortical neurons, mouse model for axonal development	Inhibits autophagy-related gene Atg12	(Yang et al., 2017; Sun et al., 2018)
miR-140	4.00	Enhancer	Human primary chondrocytes	Inhibits FUT1	(Wang Z. et al., 2018)
miR-374	5.94	Suppressor	HEK293T cells, Squamous cell carcinoma	Inhibits UV radiation resistance-associated gene UVRAG and autophagy-related gene ATG5	(Huang et al., 2012; Jing et al., 2015)
miR-223-3p	3.56	Suppressor	Experimental autoimmune encephalomyelitis mouse model, bone marrow derived macrophages, BV2 microglial cells, hepatocellular carcinoma	Inhibits ATG16L1 and FOXO3	(Li et al., 2019; Zhou et al., 2019)
miR-204	7.01	Suppressor	Rat ischemia-reperfusion model	Inhibits microtubule-associated protein 1A/1B-LC3-II	(Jegga et al., 2011; Xiao et al., 2011)
miR-34b	3.29	Suppressor	Caenorhabditis elegans	Inhibits autophagy-related Atg9	(Yang et al., 2013)
miR-217	2.98	Suppressor	Mouse glomerular mesangial cells	Targets Atg1, LC3, and Becn1	(Deshpande et al., 2018)
miR-541	3.18	Suppressor	human hepatocellular carcinoma	ATG2A and Ras-related protein RAB1B	(Xu et al., 2019)
miR-379	2.91	Suppressor	SHSY5Y cells	Lysosome-associated membrane protein 2A	(Alvarez-Erviti et al., 2013)

Pdk1, phosphoinositide-dependent kinase-1; Atg12, autophagy-related gene 12; LC3, light chain 3; PTEN, phosphatase and tensin homolog; ULK1, Unc-51 like autophagy activating kinase 1; HIF-1α, hypoxia-inducible factor 1 alpha; NDRG2, N-myc downstream-regulated gene 2; FUT1, fucosyltransferase 1; ATG16L1, autophagy-related 16 like gene; FOXO3, Forkhead Box O3; RAB1B, Rab-1B.

## Autophagy Detection and Imaging Technology

Autophagic flux, the dynamic process of autophagy, refers to the autophagophore membrane isolation, autophagosome formation/maturation, and fusion with lysosome to form autophagolysosomes (Gottlieb et al., 2015; Klionsky et al., 2016). Fluorescent LC3 fusion reporters are well suited to track autophagic flux and LC3-mediated proteolysis (Kabeya et al., 2000; Kimura et al., 2007). In mCherry-LC3 transgenic mice expressing the fluorescent LC3 under the alpha myosin heavy chain promoter, *in vivo* imaging was performed by planar imaging at 4 h after the co-injection of rapamycin (autophagy induction) and chloroquine (blocked flux). The authors reported a 23% increase in cardiac fluorescence signal compared to the baseline levels prior to rapamycin/chloroquine injection (Gottlieb et al., 2015). Autophagic flux impairment is increasingly linked to aging-associated neurodegenerative disorders (Zhang et al., 2013). GFP-LC3 transgenic mice expressing LC3 systemically, previously shown to track autophagy activation in the brain after transient middle cerebral artery occlusion (Tian et al., 2010), are well suited for intravital microscopy imaging at single-neuron resolution. GFP-LC3 punctate was seen accumulated in neurons in the ischemic hemisphere, consistent with an increase in apoptosis. GFP-LC3 overexpression did not impact neuronal aggregate clearance thus potentially allowing proteostasis during aging to be studied *in vivo*. Autophagy readout can be further combined with functional medical imaging of cerebral blood flow, a key to maintaining increased oxygen and glucose supply during healthy aging (Jahrling et al., 2015), during lifespan extension therapy such as rapamycin.

The translation of drug discovery from murine models to humans has been challenging, due to the lack of animal models that completely recapitulate the onset or the progression of CVDs. For instance, heart failure in murine models occurs acutely after surgical or pharmacological induction, whereas in human patients develops chronically over years (Strait and Lakatta, 2012; Valero-Munoz et al., 2017). The ability to carefully characterize systolic and diastolic dysfunction coupled with non-invasive imaging of autophagic flux *in vivo* thus warrants further investigation. A nanoparticle approach in apamycin-induced autophagic cardiomyocytes showed robust uptake of fluorescently labeled iron oxide Feraheme nanoparticle, due to enhanced endocytosis (Chen et al., 2013; Galluzzi and Green, 2019). Feraheme is FDA-approved thus with well-established biosafety profile in both animals and humans, possess superparamagnetic properties suitable for magnetic resonance imaging, and is amendable for further contrast agent development (Bashir et al., 2015; Yuan et al., 2018).

In conclusion, longevity treatments (pharmacological or lifestyle changes) may activate miRNA networks that can regulate progression of autophagy in the heart either as an adaptive mechanism to regulate excessive autophagy or a method for spreading of senescence. Temporal visualization of cardiac autophagy in the context of heart function is needed to ensure safety of longevity treatments.

## Author Contributions

LP and HC wrote and edited manuscript and approved final version.

## Funding

This work was supported by NIH NHLBI 1R01HL138988-01A1 (LP) and NIH K99/R00HL121152 (HC).

## Conflict of Interest

The authors declare that the research was conducted in the absence of any commercial or financial relationships that could be construed as a potential conflict of interest.
